# Myasthenia Gravis and Physical Exercise: A Novel Paradigm

**DOI:** 10.3389/fneur.2020.00675

**Published:** 2020-07-29

**Authors:** Laura O'Connor, Elisabet Westerberg, Anna Rostedt Punga

**Affiliations:** Department of Neuroscience, Clinical Neurophysiology, Uppsala University, Uppsala, Sweden

**Keywords:** myasthenia gravis, physical exercise, resistance training, neuromuscular, physical activity

## Abstract

The benefits of physical exercise for healthy individuals are well-established, particularly in relation to reducing the risks of chronic lifestyle related diseases. Furthermore, physical exercise has been seen to provide beneficial effects in many chronic diseases such as multiple sclerosis, rheumatoid arthritis, and chronic obstructive pulmonary disease and is therefore recommended as part of the treatment regimen. Myasthenia Gravis (MG) is a chronic autoimmune disease that causes neuromuscular transmission failure resulting in abnormal fatigable skeletal muscle weakness. In spite of this fluctuating skeletal muscle weakness, it is reasonable to assume that MG patients, like healthy individuals, could benefit from some of the positive effects of physical exercise. Yet exercise-related research in the field of MG is sparse and does not provide any guidelines on how MG patients should perform physical training in order to obtain exercise's favorable effects without risking disease deterioration or more pronounced muscle fatigue. A handful of recent studies report that MG patients with mild disease activity can adhere safely to general exercise recommendations, including resistance training and aerobic training regimens, without subjective or objective disease deterioration. These findings indicate that MG patients can indeed improve their functional muscle status as a result of aerobic and high-resistance strength training. This knowledge is important in order to establish collective as well as personalized guidelines on physical exercise for MG patients. This review discusses the present knowledge on physical exercise in MG.

## Introduction

Myasthenia Gravis (MG) is an autoimmune disorder of the neuromuscular junction characterized clinically by fluctuating skeletal muscle weakness and fatigue (1). Muscular weakness in MG can affect ocular, limb, respiratory, and bulbar muscles, varies over time and is often exercise induced. Physicians caring for patients with neuromuscular disorders have in the past been reluctant to actively encourage physical exercise, postulating that one could overwhelm already weak muscles with overwork (2). This “overuse weakness” (3) concern is theoretically understandable considering the exercise induced muscle weakness and fatigability seen clinically in MG, but has not been confirmed in any controlled studies. In addition, advances in modern immunosuppressive, symptomatic and supportive treatments mean that today the vast majority of well-regulated patients with MG have a good prognosis, with normal life expectancy and modest effects on activities of daily living (1).

The benefits of physical exercise for the human body and mind are unequivocal. The available evidence shows at least 20–30% risk reductions for premature mortality and chronic disease in people who exercise according to international recommended guidelines (4). More recent literature challenges earlier threshold based recommendations and shows clinically relevant benefits by simply becoming more active (4). Furthermore, time spent sedentary is an independent risk factor for all-cause mortality, cardiovascular disease, cancer, and diabetes even after statistical adjustment for the amount of deliberate exercise taken (5). Given that regular physical exercise reduces the risks for more than 25 chronic debilitating diseases including cardiovascular disease, stroke, diabetes, and various cancers (6), one would assume that a solid body of evidence showing harm would be necessary before cautioning patients with MG against reaping the benefits of physical activity.

The lack of clinical consensus guidelines on exercise for patients with MG therefore represents a conundrum for patients and caregivers alike (7). Patients want to know if they can exercise safely, what kind of exercise they should perform and how it will affect their disease.

This review describes the few studies that exist on the topic of physical exercise and MG, thereby informing patients and clinicians seeking to establish physical exercise routines, and providing a base on which to guide the necessary development of future larger randomized controlled trials.

## The Effects of Exercise on Autoimmune and Neuromuscular Diseases

Autoimmune disorders represent a wide range of heterogenous chronic diseases caused by failure of the immune system to distinguish self from non-self and mounts therefore an immunologic response against the body's own tissues. Physical exercise is considered safe in many autoimmune diseases including for example systemic lupus erythematosus (SLE), rheumatoid arthritis (RA), multiple sclerosis (MS), and inflammatory bowel disease (IBD). In several of these autoimmune conditions, physical exercise is even an established part of the treatment regimen (8). As a general trend, patients with autoimmune conditions have lower physical activity levels than the general population (8). The incidence of RA, MS, IBD, and psoriasis is also higher in people who are less physically active (9–11). Advances in biological treatment of autoimmune conditions have improved quality of life (QoL) for many of these patients, however self-modifiable lifestyle factors also play an important role in patients' well-being and immune system function.

Physical exercise leads to an immune response, with a rise in T regulatory cells, decreased immunoglobulin secretion, and a shift in the Th1/Th2 balance toward decreased Th1 cell production (8). In addition, physical exercise causes release of the myokine (cytokine released by skeletal muscle) IL-6, which induces an anti-inflammatory response through IL10 secretion and IL-1β inhibition. Additional beneficial effects of physical activity are improvement in mood, reduction in fatigue, and positive effects on cognition and mobility, seen for example in patients with MS (8). Furthermore, physical activity improves QoL and reduces co-morbid cardiovascular disorders in SLE and RA patients (8).

General fatigue and cardiovascular deconditioning are more prevalent amongst patients with neuromuscular diseases compared with the general population (12). Regarding neuromuscular disorders in general, few well-designed studies have been conducted on the benefits or disadvantages of physical exercise (12). In inflammatory muscle diseases, including polymyositis and dermatomyositis, exercise enhances aerobic capacity, improves muscle function, and reduces disability (13). In patients with inflammatory polyneuropathy, significant improvement in muscle resistance, functional activities, and physiological adaptations following exercise are reported (14). In addition, reduction in chronic fatigue has been reported in patients with facioscapulohumeral muscular dystrophy type 1 (15).

A detailed review on exercise in relation to a broad spectrum of neuromuscular diseases concluded that a regular exercise regimen is beneficial in neuromuscular disease, whether aerobic/endurance or strength/resistance training (16). This review recommends that patients should establish an exercise program with their physician and that those with neuromuscular junction disorders and metabolic myopathies should combine strength training and submaximal aerobic exercise on alternating days (though it is unclear exactly what evidence this was based on), aim to slowly increase the number of repetitions and achieve 65% of maximal heart rate (220-age/min) during aerobic training.

## Challenges of Measuring Fatigue in Myasthenia Gravis (MG)

One challenge regarding physical exercise evaluation in MG is the objective measurement of fatigue. Several of the studies mentioned in this review fail to define fatigue and there is no standard terminology. Fatigue involves both performance fatigability and fatigue perception, and is a distinct primary symptom which must be differentiated from pre-existing muscle weakness (17). A unified taxonomy of fatigue has been proposed for neurological diseases, which may be useful in future research studies to differentiate between performance fatigability and fatigue perception (18). Fatigability is defined as performance decline during prolonged cognitive and physical activities and muscle fatigability is an objective decline in strength as the routine use of muscle groups proceeds (19). In the early stages of MG, impaired neuromuscular transmission causes muscle weakness, physical exhaustion, and tiredness and this is evidenced by pathological decrement at neurophysiological evaluation with repetitive nerve stimulation (RNS). However, routine clinical MG score and RNS are suboptimal at detecting fatigue in proximal muscles (20) and the persistence of fatigue in patients with stable longstanding generalized disease who lack muscle fatigability on bedside testing requires deeper explanations. The quantitative MG score (QMG) encapsulates fatigability in the early stages of disease by measuring endurance over a fixed time period of for example outstretched arms and legs and neck flexion. However, neither QMG nor RNS are sufficiently sensitive to capture fatigability in longstanding stable generalized MG (17). The Fatigue severity scale (FSS) has been employed in some studies (21, 22), however this was developed primarily for use in MS and SLE and may not reflect the fatigue of MG, which is commonly of a different nature. Self-report fatigue scales can broadly be classified as measuring perceptions of fatigue. Nevertheless, recent literature suggests that research questions regarding fatigue may best be assessed using multiple measures (18). Also, one should take into account other disease unrelated potential confounders, for example body mass index (BMI) which can be a confounder for the presence of fatigue.

Functional evaluations include the 6-min (6MWT) and 2-min walk tests. The 6MWT is an established simple assessment tool for aerobic capacity and endurance and represents a submaximal test of exercise capacity. It measures the maximum distance a patient can walk in 6 min, oxygen saturations and pulse are often also monitored (23). The shortened version of the 2 min walk test has recently been described as a valid alternative to describe walking capability in patients with neuromuscular diseases and is chosen in patients who cannot complete the 6MWT due to fatigue or dyspnoea (24). An evaluation of the reliability of these tests was conducted on 31 patients with MG (MGFA class II or III) (25). On the first admission for testing, timed walk tests were performed at 3-h intervals on the same day, 1–2 h after pyridostigmine intake. Three to seven days later patients were admitted for a second time and the tests were repeated in the same fashion. Both timed walk tests were found to be reliable between test and retest conditions and they had good construct validity. They performed similarly in their ability to reflect which MGFA severity class (II or III) the patients fitted into. The study also noted the difficulty in practically measuring maximum oxygen consumption in a clinical setting (25). Other potential confounding factors which may influence result analysis in these types of exercise studies are medication effects, for example corticosteroids could mask the antihypertensive benefits of exercise and can induce weakness due to steroid myopathy.

## Fatigue, Fatigability, and Deconditioning in MG

Exercise capacity in MG may be restricted by proximal muscle weakness, fatigability, and impairment in respiratory muscle function (26). Furthermore, the inherent muscle weakness and the subsequent risk of increased sedentary behavior in MG may in turn increase the risk of becoming overweight, developing respiratory infections, and osteoporosis which in turn leads to falls and fractures (1). Poor physical fitness in healthy individuals as well as MG patients may result in a “vicious circle” where physical deconditioning causes lethargy and fatigue (27) and in younger individuals, non-specific fatigue disorders are part of the differential diagnosis for MG (1). In the child and adolescent population, the objective measurement of fatigue is complex and there are no guidelines on how much exercise children and adolescents with MG can and should take, representing a conundrum for the patients themselves, their parents and physicians. Bearing this in mind, it is intriguing how little is known about baseline fitness and conditioning levels in MG patients. One study monitored the baseline activity patterns of 27 MG patients with mild to moderate MG (13 female, mean age: 62 years) using an accelerometer worn consecutively for 7 days (28). Amounts of moderate and vigorous intensity activity were measured in terms of metabolic equivalent of task minutes (MET-min), physical activity level (PAL), number of steps/day and sedentary time, and the results were compared with the American College of Sports Medicine guidelines for exercise (28, 29). Participants were found to be engaged in sedentary activity 78% of their time and reached a mean number of 7,462 steps/day, with only 22% (all women) achieving the recommended level of 10,000 steps/day. Despite this, when all types of moderate and vigorous activities of at least 10 min duration were counted for, 78% still achieved the minimum average physical activity output of 64 MET min/day as recommended by the American Heart Association (30). The results regarding time spent sedentary mirror findings in the general population, showing undesirably long periods of sedentary time despite deliberate efforts to take exercise. In comparison to data on healthy individuals the MG patients were less physically active and were more often sedentary. MG disease severity as measured by MG Composite score (MGC) interestingly did not correlate with any of the different measures of physical activity. This lack of correlation emphasizes the complexity of factors which can lead to sedentary behavior, including disease perception of the individual with MG and their physician.

Objective measures of neuromuscular function that reflects the entire motor unit include compound motor action potential (CMAP) (31), which differs between trained and untrained individuals, and neuromuscular ultrasound. However, structural measures of exercise effects are not well-established areas of extensive evaluation, and in relation to neuromuscular disease they have not been previously studied (31–33).

One study which focused specifically on fatigue and fatigability in MG patients, assessed the time dependent physical performance of 32 individuals with stable generalized MG and compared with 17 healthy controls (17). A gradual performance decline within a given time period was proposed to be suitable for quantifying fatigability in MG patients, especially in those without neuromuscular deficits on routine clinical assessment. The MG patients had low QMG scores and no pathological decrement on RNS, indicating stable disease. Both patients and controls were assessed with the 6MWT and the arm movement test (AMT), where subjects hold a weight with the arm horizontally outstretched and move it between 2 points. Fatigability was assessed by performing these repetitive movement tasks of proximal muscles and calculating a linear trend to reflect fatigability. Subjects also filled out fatigue questionnaires to assess fatigue perception including MG fatigue scale, MG activity of daily living scale, MG quality of life (MGQoL), Pittsburgh sleep quality index, and center for epidemiological studies depression scale. In MG patients the mean value for the linear trend for both AMT and 6MWT was negative, indicating that a gradual decrease in performance was an objective parameter of fatigability even though only two out of 32 patients had mild pathological decrement in RNS of the trapezius muscle after AMT exercise. Controls had a positive linear trend, indicating physiologically stable performance. Interestingly, the performance decline in AMT correlated with current elevation in AChR antibodies and was not correlated with BMI. The perception of physical fatigue was significantly higher in MG patients than controls, although there was no correlation between the subjective fatigue parameters and the objective linear trend results. This underlines the multifactorial etiology of fatigue and reinforces the importance of considering psychological and lifestyle factors in patients instead of always increasing the dosage of acetylcholinesterase inhibitors in patients experiencing worsened fatigue.

Another pilot study investigated whether a combination of psychological and physical therapy could reduce fatigue in stable MG patients (22). Nine patients completed a 10-week program involving breathing and relaxing exercises, muscle stretching exercises, and teaching sessions on the management of stress and fatigue. Fatigue was measured using the modified fatigue impact scale (MFIS), the visual analog fatigue scale (VAFS), and the FSS at the start of the study, at various intervals during the study and 3 months after study completion. There was a slight improvement in the physical and psychosocial subscale of the MFIS during the program and a significant improvement in the VAFS at the end of the program, with no improvement in FSS. The improvement was minor and unsustained with all fatigue scores returning to baseline 3 months after study completion.

## Clinical Trials on Physical Exercise in MG: Challenges and Shortcomings

The available studies on MG and physical exercise are few, with methodological shortcomings mainly due to small sample sizes, MG being a rare condition, which further limits subgroup analyses according to age, gender, MG severity, and subtype. Furthermore, the studies cannot be directly compared with each other due to the different methodologies employed.

Most training intervention studies to date have relatively short training and follow-up periods, although the health and strength benefits of an exercise intervention are transient unless patients keep up their physical training. Muscular adaptions in response to strength training such as muscle fiber hypertrophy and increased protein synthesis take 6–8 weeks to develop. On the other hand, neural adaptions such as increased activation and motor unit synchronization occur as early as 2 weeks and account for early strength gains during a training program (3). Cross transference is a phenomenon observed whereby neural adaptation in a trained single limb causes strength increases in the contralateral limb, and must be considered when reviewing studies where the design involves one limb being trained and the contralateral limb acting as a control (3). However, longer interventions and follow-up times could lead to higher numbers of dropouts due to the time-consuming nature of the intervention, and time for more confounding factors coming into play. A control group is desirable, but it is challenging to design a comparable group of healthy individuals in relation to exercise.

There is moreover an obvious inability to double blind patients to exercise as an intervention.

Which outcome measures to choose in exercise studies on MG patients is far from clear. There are general recommendations regarding evaluation of MG disease severity (34). However, when it comes to measuring functional outcome and physical improvement there are a huge number of measurements of varying potential significance to choose from, none of them having been evaluated for MG patients. Therefore, previously performed studies on physical exercise in MG often have used different outcome measures (as presented below), making them difficult to compare.

Outcome measures such as fatigue and QoL are inherently subjective and also fluctuate.

A flaw in many studies is the use of strength or force as an outcome rather than functional performance, which may be a more useful indicator of benefit in neuromuscular disease (12).

Despite a variety of modalities being available for the objective evaluation of functional exercise capacity, there is no “gold standard” physical performance test in clinical practice, rather one must choose between a high tech complete assessment of all systems involved in exercise performance or a simpler more practical test, based on the clinical question at hand (23). Similarly, the benefits of physical exercise can be more difficult to quantify than in healthy individuals given the fluctuating nature of muscle weakness characteristic to MG.

## Potential Influence of MG Treatment, Comorbidities, and Aging for Physical Exercise

Most patients with mild to moderate MG are treated with symptomatic treatment, acetylcholinesterase inhibitors (AChEIs) and/or low dose of corticosteroids (e.g., prednisone). When this medication fails to clinically stabilize their MG, corticosteroid sparing agents are used, including azathioprine, cyclosporine, mycophenolate mofetil, and in more severe cases rituximab and other medications are used (35). Unfortunately, most of the studies on physical exercise and MG described below fail to clearly describe the effects of medications and comorbid conditions on these patient's ability to perform exercise. In particular, glucocorticoids (e.g., prednisone) have a range of effects which may interfere with an MG patient's ability to perform exercise. These medications are associated with weight gain, partly due to increased appetite and redistribution of body fat causing Cushingoid appearance even at low doses (36). Furthermore, glucocorticoids are associated with a variety of adverse cardiovascular effects, including fluid retention, premature atherosclerotic disease, and arrhythmias. Cardiovascular disease risk is dose-dependent and may be low or absent in patients on low-dose glucocorticoid therapy (37), whereas hypertension is a poorly understood dose related adverse effect of glucocorticoids (38). Glucocorticoids cause increases in serum glucose levels, although the development of *de novo* diabetes in a patient with initially normal glucose tolerance is rare (39). Other co-morbidities related to the side effects of corticosteroid treatment are osteoporosis and less commonly dose-related myopathy and neuropsychiatric conditions. MG patients with established osteoporosis should continue with weight bearing exercises to prevent bone loss and muscle atrophy in combination with bone protecting medications in those taking long term glucocorticoids. The more uncommon side effect of myopathy impairs exercise tolerance and could be initially mistaken for a worsening of the patients MG status. Additionally, physical exercise has a well-established role in reducing weight gain and improving cardiovascular risk profiles, which may help to negate some of these cardiovascular side effects in MG patients. Physical exercise also has beneficial effects on mood which may help to combat the neuropsychiatric side effects of glucocorticoids.

The symptomatic medication pyridostigmine is a parasympathomimetic reversible cholinesterase inhibitor, which enhances the efficiency of cholinergic transmission at the neuromuscular junction. Rare side effects include transient bradycardia and hypertension. However, to our knowledge, no evidence exists to caution patients with cardiac disease against exercise in combination with AChEIs. Rather, in non-MG patients with cardiovascular disease pyridostigmine delays the onset of myocardial ischaemia by inhibiting the submaximal chronotropic response to exercise on a treadmill (40). Furthermore, in non-MG patients with chronic heart failure, the effects of pyridostigmine on parasympathetic tone lead to improved heart rate recovery 1 min after exercise (41).

Azathioprine and mycophenolate mofetil have a wide range of side effects including gastrointestinal upset, bone marrow suppression and possible increased susceptibility to infection and malignancy; however, to the best of our knowledge these drugs have no direct effects which would contraindicate physical exercise.

Coexisting conditions are rather common in MG and must therefore be considered (35). Many patients suffer from comorbid cardiac disease, and the benefits of exercise for cardiovascular health are unequivocal. Approximately 15% of MG patients have a second autoimmune disease (35). Thyroid disease is the most common coexisting condition, and the effects of exercise on circulating thyroid hormone values remains controversial (42). SLE and RA are the next most common disorders. Therapeutic exercise programmes appear to be safe in SLE patients and result in improvements in physical fitness, fatigue, and depression (43). In RA patients, exercise has been seen to be effective in improving disease related outcomes, including functional ability and systemic manifestations, such as the increased cardiovascular risk (44).

In addition, normal aging in MG patients and an already high age in late onset MG patients, can naturally give rise to difficulties exercising. Although many of the studies on MG patients considered below included some patients over 70, none were targeted at an older population. One study employed balance strategy training in MG patient, exercises that address the functional needs of patients, which has been shown to be beneficial in the elderly (45). The results from this study were positive but the study only included one patient over the age of 70. Studies of exercise in frail older adults without MG have shown beneficial effects, although the optimal program remains unclear (46).

## Active Physical Exercise Intervention Studies in MG

In regard to studies with a primary focus on exercise interventions in MG patients, the literature comprises of only a handful of studies and these are therefore considered in relative detail below, followed by a summary (Table 1).

**Table 1:** Summary of active physical exercise intervention studies in myasthenia gravis (MG).

**Study**	**Design**	**Aim**	**Participants**	**Training protocol**	**Results**
Rahbek et al. (47)	2 arms randomized+ stratified 20 supervised training sessions over 8 weeks	1. Feasibility of AT and RT 2. Muscular strength, oxygen uptake, functional capacity, psychological well-being	15 in total MGFA class II+III 3 dropouts (1 = worsened bulbar symptoms, 2 = other unrelated reasons) 6 completed AT 6 completed RT	• AT protocol (6 patients, moderate to high intensity AT): Incremental cycle test to exhaustion 75–80% of max HR by 8 weeks • RT protocol (6 patients): Full body progressive resistance exercises e.g., weighted step ups, bench press Progressive increase in reps	• Exercise feasible for most patients with mild MG-over 90% adherence to protocol • Improved knee extensor+shoulder abductor strenth, 30SSS, BBT, less fatigability of knee extensors in RT group • Stable V02 peak in both groups • Deterioration in MGQoL score in AT group
Westerberg et al. (48)	Prospective pilot study. Tailored, supervised AT+RT twice weekly over 12 weeks	Safety and efficacy of physical exercise training in MG	10 in total MGFA class I+II 8 AChRAb+ 2 AChRab-	• AT protocol-bicycle interval training • RT protocol-8 resistance exercises e.g., biceps curl, sit ups max 2 sets of 10 reps • Balance training	• All patients completed the program • No change in disease activity (MGC and RNS decrement) • Improved 6MWT and 30SSS • No change in muscle force (dynamometer) • Increased muscle+reduced fat mass • Subjective improvement (ESES) • Serum-significant decrease in disease specific miRNAs
Westerberg et al. (21)	Non-blinded observational study. Supervised AT and RT over 12 weeks	Safety and efficacy of physical exercise training in MG Effects of exercise on functional muscle parameters	14 in total MGFA class I-IV 3 dropouts (all unrelated) 8 AChR Ab+ 1 Musk Ab+ 2 AChR/MuSK- 5 EOMG 6 LOMG	• AT protocol-bicycle interval training • RT protocol-7 resistance exercises e.g., rowing, • Biceps curl, sit-ups max 2 sets of 10 reps	• No clinical MG deterioration, MGC and QMG slight decrease, no RNS decrement deterioration • Majority exceeded 70% of pulse max during training • 10 increased resistance weights on 4 of 7 strength exercises • 4 increased their bicycle resistance • 30SSS improved • Muscle thickness increased in biceps and quadriceps (CMAP amplitude, neuromuscular ultrasound) • 12MWT, TUG, handgrip strength unchanged • MGQ0L15 improved • FSS+ESES unchanged
Wong et al. (45)	Pilot study 16 session workstation intervention training 1–2 times/week	Improve balance and functional mobility in MG patients	7 in total	• Balance strategy training-16 tailored balance strength and endurance exercises e.g., heel to toe walking, sit to stand, ball catching, and throwing	• Clinically significant improved QMG score • Clinically significant improved TUG, partially maintained at follow up
Lohi et al. (49)	Pilot study 10-week training period	Determine whether MG patients can increase muscle force or resistance to fatigue with physical training	11 in total aged 25–50 mild to moderate severity MG	• Subjects randomized to dynamic strength training of right arm and left leg or vice versa, contralateral extremity = within subject control • Muscle fatigue assessed using peak values achieved during reps of 3 s max contractions	• 9 could not complete all reps in each set and 8 could not increase workload as planned • 23% improvement in maximal voluntary muscle force (dynamometer) compared with 4% on the untrained side • Inconclusive results of fatigue test
Lucia et al. (50)	Case report 3-month training program 5 sessions weekly	Restore the subject's capacity for independent living by improving exercise tolerance and limb weakness	29-year-old female with MG (diagnosed age 24) and McArdles disease since childhood and obesity AChR Ab+, thymoma	• Low to moderate intensity AT walking, cycling or swimming increasing duration from 10 to 60 min Carbohydrate ingestion to prevent rhabdomyolysis	• Regained ability to live independently • Increased exercise time by 44% • Increased V02 peak by 50% • Increased peak heart rate and peak workload (watts) post intervention • Subjective improvement in well-being and ability to perform ADLs

*AT, aerobic training; RT, resistance training; 30SSS, 30 second sit to stand; BBT, box and block test; MGQoL15, MG quality of life-15; MDI, major depression inventory; MFIS, modified fatigue impact scale; MGC, MG composite scale; ESES, exercise self-efficacy scale; Reps, repetitions; EOMG, early onset MG; TUG, timed up and go; LOMG, late onset MG; QMG, quantitative myasthenia gravis score; ADL, activities of daily living*.

In a two arm randomized and stratified study by Rahbek et al. (47), on the feasibility of exercise training in MG, 15 patients were randomly assigned to either a progressive resistance training (RT) or an aerobic training (AT) intervention over 8 weeks. Six MG patients completed the RT program and six patients completed the AT program. The primary outcome was feasibility of these two types of training, based on adherence dropout rates and adverse events, and secondary outcomes included increases in muscular strength, oxygen uptake (VO2 max), and various functional capacity measurements such as 6MWT. Patients completed three sessions with a battery of tests; *before run in* to familiarize themselves, *before intervention*, and *after intervention*. Patients continued on their routine medications throughout the study and the three test sessions were conducted at the same time of day and 1 h after pyridostigmine intake to reduce diurnal variation and fluctuation in medication effects. A focused 25 repetition isokinetic fatigability test of knee extensors was also performed unilaterally on the dominant side in order to assess the muscular fatigability of the subjects.

The training programs are described in Table 1. In terms of feasibility, the moderate to high intensity level of the exercise program did not appear to deter participants, with over 90% adherence rates observed, in line with adherence rates often reported in studies on exercise interventions in MS (51). Three patients (20%) dropped out during the intervention which is in line with dropout rates observed in exercise studies of people with neurological disorders and healthy individuals (33). One patient dropped out due to bulbar symptoms requiring prednisone 4 weeks into the study, while the other two dropped out due to reasons unrelated to the study. The dropout who deteriorated clinically may have worsened due to the training, but it is also possible that this deterioration began before the exercise intervention, as the individual was noted to have an increase in QMG score from 2 to 4 primarily in the categories of speech and facial muscle scoring in the lead up to the intervention. Both groups of patients reported adverse events including temporary worsening of fatigue and bulbar symptoms, but this did not affect participation except for the dropout described above who dropped out from the RT group.

The psychological tests of MGQoL15 questionnaire, major depression inventory, and modified fatigue impact scale showed lower scores in the RT group after the intervention, indicating no negative effects. However, in the AT group there was a significant deterioration in MGQoL15 score and scores almost doubled across all three psychological tests (where higher scores indicate worse symptoms), although this was not statistically significant. The authors suggest that rise in core temperature may have worsened symptom severity in the patients, as has been seen before in studies of AT vs. RT in MS patients (52, 53). While no adverse effects on the clinical status of the patients were noted, this observation should be examined further in future studies.

In terms of secondary outcomes, the RT group showed improved muscle strength post intervention but not the AT group, as expected. The 10% increase in maximal knee extensor strength seen in the RT group may have clinical relevance as deficits in knee extensor strength have been described in MG patients (54). There was a 23% increase in shoulder abductor strength in the RT group, the shoulder abductors being the most affected muscle group in MG patients (55). The study was unable to demonstrate increase in the VO2peak test (which measures maximum oxygen uptake during incremental exercise and is a quantitative measurement of aerobic fitness) in either group. This was surprising as previous AT interventions in neuromuscular disease have given rise to improvements in aerobic capacity (12), and a case report on exercise in MG increased aerobic capacity (50). Improved 30-second-sit-to-stand test performance (30SSS), which measures functional leg strength and box and block, which measures manual dexterity was seen in the RT training group post training intervention, almost reaching the minimal level considered necessary for clinical difference in neurological disorders (50, 56). The fatigability test of knee extensor muscles revealed intriguingly less fatigability of these muscles post exercise intervention in the RT group.

In summary 8 weeks of moderate to high intensity AT and PRT were feasible for most patients with mild MG. Secondary outcomes revealed improved muscle strength and functional capacity in the RT group, whereas the AT group did not show these improvements.

In a prospective pilot study by Westerberg et al. (48), 10 MG patients performed supervised AT and RT twice weekly for 12 weeks. Patients were examined at the same time of day before and after the study to reduce the effects of diurnal variation. These patients followed exercise guidelines recommended for healthy adults, 150 min of medium intensity aerobic exercise weekly and strength training twice a week (30) under physiotherapist supervision. Participants completed tailored 90-min training programs as described in Table 1.

After 12 weeks there was no change in disease activity, as measured by MGC, and RNS decrement remained unchanged. None of the patients discontinued the training program due to increased muscle fatigue, and peak expiratory flow rates remained constant.

A significant improvement was seen in the physical performance measures of 6MWT and 30SSS. Improvement in the function of proximal muscles was seen in the form of an enhanced ability to bear increasing weights with these muscles. Furthermore, increased CMAP amplitude, which correlates with isometric muscle strength (31), was seen in the biceps and quadriceps muscles. There was however no significant change in muscle force as measured by hand held dynamometer and handgrip strength test, maximum repetitions of toe rise endurance test and balance as measured by time in Romberg's test. Increased muscle mass and reduced fat mass was seen in the subjects, with no significant change in BMI. Pulse (% of max) remained consistent in the patients over the course of the training period, despite a gradual increase in bicycle load resistance, demonstrating positive aerobic effects of the training program.

Patients also subjectively reported improved ability to perform physical training after completion of the training program on the exercise self-efficacy scale. Serum analysis in the patients revealed a transient rise in CKMB and myoglobin after exercise without reaching abnormal levels, it remains to be elucidated whether this transient rise represents muscle damage or rather disruption in energy control processes at a molecular level (57). Intriguingly, a significant long term decrease was observed in the disease specific micro RNAs miR-150-5p and miR-21-5p, which have been proposed as potential MG biomarkers (58).

A prospective unblinded observational study by the same group (21) recruited 14 MG patients to a similar 12 week aerobic and resistance strength training program involving cycling and strength training. Eleven patients completed the program, with three dropouts, none of which discontinued due to MG deterioration. This study examined the effects of exercise on disease activity but also focused on the effects of physical exercise on functional skeletal muscle parameters in the participants. Medications were unchanged during the training period, except for three patients who were able to lower their doses of acetyl cholinesterase inhibitors during the training period.

The participation rate of the 11 participants who completed the study was between 75 and 96%. The vast majority of patients exceeded 70% of pulse maximum during training periods with high resistance loads. Ten participants increased their resistance weights in at least four of the seven strength training exercises and eight patients increased their bicycle resistance in the second half of the training period.

The clinical markers of disease activity, median MGC and QMG scores, decreased slightly during the training period, indicating slight improvement in MG status, and no patients described any subjective negative effects of the training program. MG specific quality of life assessment MGQOL 15 tended toward higher scores i.e., improved quality of life however not significantly. There were no significant changes in FSS or on the subjectively measured exercise self-efficacy scale, which was scored highly before beginning the intervention. RNS did not deteriorate after the 12-week-program, one patient having abnormal decrement after the program compared with four patients before, and respiratory muscle function as measured by peak expiratory flow remained unchanged. BMI, blood pressure, resting pulse, and body composition fat vs. muscle mass did not change significantly. The physical performance-based measure of 30SSS improved. Twelve-minute-walk-test, timed-up-and-go, which assesses mobility and falls risk, and handgrip strength tests remained unchanged. An improvement was noted in the functional muscle measures of isometric muscle force as measured by hand held dynamometer recording from the biceps brachii and quadriceps muscles. Muscle thickness increased as measured by CMAP amplitude and neuromuscular ultrasound increased in the rectus femoris muscle.

Serum analysis revealed a modest few changes; a significant increase in apolipoprotein A1 levels, plasma-25-hydroxyvitamin D decreased significantly with unchanged calcium, and phosphate and HbA1c levels were non-significantly lowered. All functional outcome measures of the proximal leg muscles improved in this study, with no improvement seen for arm muscles. This arm leg difference remains unexplained, though it was noted in one previous study (59) and may have been influenced by the female preponderance in these two studies.

While this study shares the common shortcomings of small sample size and short intervention time, beneficial effects on subjective, and objective muscle outcomes especially in proximal leg muscles were noted. There was no evidence of clinical MG deterioration in these subjects with well-controlled MG.

In a pilot study specifically aimed at improving balance and functional mobility in MG patients, seven MG patients underwent a 16 session workstation intervention, completing 1 or 2 sessions per week (45). Balance strategy training is based on exercises that address the functional needs of patients by targeting the function of neural sensorimotor processes involved in postural control (45). These exercises have been shown to increase balance strength and functional ability in several populations, particularly the elderly as postural instability increases with age due to deteriorating function of dynamic sensorimotor processes and cognitive processing (60). Subjects performed a total of 16 tailored balance strength and endurance training exercises, training once or twice a week according to their ability. The training regimen was developed to address the functional needs of subjects, has been shown to be of benefit in osteoporosis and falls prevention and involves a range of exercises such as heel toe walking, sit to stand, ball catching, and throwing (45). The intervention resulted in a clinically significant improvement in QMG core >15% in subjects post intervention and additional improvements at follow up. A clinically significant improvement was seen in the timed up and go test post intervention which was partially maintained at follow up and reflected improvements in dynamic balance and functional ability. The distance mobilized in the 6MWT increased, but this was not statistically significant.

In a study published in 1993, 11 MG patients with mild to moderate MG underwent a strength training program of ~30 sessions over 10 weeks (49). Six patients had mild symptoms, two had moderate symptoms from the limbs and three had mostly ocular and bulbar symptoms. Eight patients were medicated with acetylcholinesterase inhibitors and these were tested at a fixed time after the last dose intake. Subjects were randomized to dynamic strength training using weights of either their right arm and left leg or vice versa, the contralateral extremity serving as a within subject control.

Voluntary maximal muscle force was measured in three muscle groups: those involved in knee extension, elbow flexion, and extension. Muscle fatigue was assessed using the peak values achieved during repetitive three second maximal contractions. The subjects experienced slight muscular pain during the run-in period as to be expected but none complained of adverse effects during the training period. However, nine MG patients could not complete all 10 repetitions in each training set and eight patients could not increase their work load as planned. Six patients managed well-training elbow flexion, the remaining four having trouble with the number of repetitions and increasing work load. Only one patient was unable to use the initial predicted training weight for knee extension but managed well-later, as did all the others. The results showed a significant 23% increase in maximal voluntary muscle force in knee extension compared to 4% on the untrained side. All patients reported subjectively that they improved their strength and resistance to fatigue during the training period. The fatigue test employed showed large test-retest variability and most subjects experienced muscle pain after testing. The authors concluded that dynamic training with small loads is relatively well-tolerated and provides some improvement in strength in patients with mild MG.

The largest randomized controlled trial (RCT) to date on the subject is ongoing and plans to evaluate the benefits of a home-based physical exercise program compared to usual care in 42 MG patients with stable disease (61). This multicentre interventional single-blinded two arm parallel group RCT will see patients aged 18–70 years undertake a 40-min home-based exercise program using a rowing machine 3 times a week for 3 months as an add-on to usual care. Patients will be observed for 3 months prior to commencing the intervention and followed up for 3 months after completing the intervention. The control group will receive usual care without the addition of the exercise intervention. The primary outcome is mean change in MGQoL and secondary outcomes include measures of functional limitations e.g., MG activities of daily living scale as well as clinical scores and measures of respiratory function, muscle force, fatigue, anxiety, and depression.

Another interventional trial to be completed in 2020 (NCT01047761) aims to characterize the fitness level and cardiovascular disease risk profile in 30 generalized MG (GMG) patients and determine whether a 3-month moderate intensity home exercise program is safe and provides benefits in deconditioned stable MG patients. The objective is to investigate whether the exercise program can reduce cardiovascular risk and improve physical activity levels, strength and fitness. Primary outcome measures are cardiovascular fitness, gait and physiological reserve. Secondary outcomes are ambulatory function as measured by 6MWT and accelerometer, muscle strength (dynamometer), MGQoL, QMG and pulmonary function tests. Volunteers will undergo 3-month home-based exercise intervention 3 days a week of progressive intensity involving aerobic training (walking), resistance training and breathing exercises.

Of interest, a study on physical exercise in MG with focus on patients undergoing thymectomy concluded that exercise is not a contraindication in MG, and rehabilitation can be safely performed before and after thymectomy, reducing operative risks and decreasing recovery time (62). Forty-six MG patients who underwent thymectomy for MG during the years 2005–2010, completed pre- and post-operative rehabilitation programs and were matched with a “control patient” who underwent thymectomy without preoperative rehabilitation (control patients were retrospectively chosen from within 5 years preceding the active study). The program involved aerobic training, mild resistance training, and pulmonary rehabilitation. All patients but two completed the program and those completing the program had reduced operative risk, decreased early postoperative morbidity, lower rates of admission to intensive care, and shorter hospital stays. Measures of disease activity such as QMG score as well as 6MWT and forced vital capacity (FVC) measured a significantly faster recovery at 3 months. There was however no significant difference in complete stable remission.

## MG and Sports Participation

There is a paucity of information on MG patients and sports participation and no clear guidelines for athletes with MG (7, 63, 64). To our knowledge, four case reports exist on athletes with MG and are summarized in Table 2. In addition, one case report (referred to in Table 1) describes a 29-year-old lady with both MG diagnosed at age 29 and Mc Ardle's disease (muscle glycogen phosphorylase deficiency) since childhood, who showed a significant increase in her exercise capacity and a regained ability to live independently after completion of a 3 month aerobic exercise training program (50).

**Table 2:** Summary of available case reports on MG and sports participation.

**Author**	**Case description: clinical status, medication**	**Type of sport**	**Outcomes**
Birnbaum et al. (63)	36-year-old female; GMG: MGFA II B; AChR Ab+ Pyridostigmine, Azathioprine (100 mg daily)	Marathon running Trained 5–10 km weekly runs during the first 2 years after MG onset	• Stable MG status i.e., persistent right-handed weakness, occasional ocular, and bulbar symptoms at the end of pyrodistigmine dose • Discontinued azathioprine • Normal respiratory function • Stable limb strength in knee flexors and extensors • Improved MGQoL
Scheer et al. (65)	55-year-old male; GMG: MGFA IIA diagnosed 5 years prior to race Pyridostigmine 60 mg 6 times daily; Prednisone 10 mg daily	Ultramarathon in a hot environment 35°C ambient temp	• Completed 220 km ultramarathon (his 5th ultramarathon) • Fluctuating leg weakness, dysphasia, dysphagia, and dyspnoea during the race which subsided with rest or pyridostigmine
Stout et al. (66)	26-year-old male; GMG Running, baseball, weightlifting athlete Pyridostigmine 60 mg daily; Azathioprine 150 mg daily; Prednisone 60 mg daily	15-week resistance training program e.g., bench press, leg curls +creatinine supplementation	• Increased body weight • Increased fat free mass • Increased peak strength for leg extension and leg flexion
Leddy et al. (67)	17-year-old male; GMG (MGFA IIA), AChR Ab– Prednisone discontinued by the patient himself-	Collegiate football player Continued to train football while experiencing mild left ptosis and mild decrease in tolerance to intense exercise	Retired from football with a back injury, continued to play recreational sports

*GMG, generalized myasthenia gravis; AChR+, acetylcholine receptor antibody seropositive; AChR-, acetylcholine receptor antibody seropositive; MGQoL, Myasthenia Gravis quality of life 15 score*.

## Respiratory Muscle Training in MG

Respiratory insufficiency due to weakness of the diaphragm can be a threat to patients with GMG (35). Patients with GMG often have restrictive spirometry and may exhibit a “myasthenic pattern” of decremental respiratory volumes during maximal voluntary ventilation (68). They may also demonstrate obstructive spirometry, with lower FEV1/FVC ratio than controls, even in well-regulated disease (26). Patients may complain of dyspnoea on extreme effort due to muscle weakness, and ventilatory muscle impairment impairs physical activities and patients' activities of daily living due to perceived fatigue. Level III evidence (indications of effectiveness) exists for the benefits of breathing exercises for MG patients (69).

One study randomized 27 stable MG patients into training and control groups in an 8-week- intervention. The training group participated in training of diaphragmatic breathing and pursed lips breathing and improved their respiratory muscle endurance, maximum inspiratory and expiratory pressures, and thoracic mobility in comparison with their own baseline levels and compared with controls (70). Smaller studies have shown the benefit of long-term respiratory muscle endurance training on lung function and respiratory endurance in mild to moderate MG (71, 72).

A recent cross-sectional trial showed that expiratory muscle strength is also a predictor of functional exercise capacity in GMG (73). Twenty-eight GMG patients (15 women, median age 53.5 years) of MGFA class II-III were tested with 6MWT, pulmonary function tests, respiratory strength and endurance assessment. Nearly 40% of the patients had expiratory muscle strength (as measured by maximal expiratory pressure) below the lower limit of normal. Multiple linear regression analysis revealed that the percentage of predicted expiratory muscle strength was a significant and independent predictor of the achieved percentage of predicted 6MWT distance (according to age and gender).

Despite speech difficulties being a symptom of MG, to our knowledge there is a paucity of evidence on the benefits of tailored exercise regimens to improve speech in these patients. One case report of a patient whose dysphonia contributed to the diagnosis of MG showed benefit from drug therapy combined with speech therapy, which improved voice quality and great impact on quality of life (74).

## Conclusion

Existing research on exercise in patients with MG is limited in scope. Despite infrequent cautionary observations that arise from the literature, such as the single patient who dropped out due to deteriorating bulbar symptoms and the deterioration in MGQoL observed in the AT group of the Rahbek study (47), the conclusions are overwhelmingly positive in favor of the benefits of exercise in MG. Clinically stable MG patients, just like healthy individuals, should be able to reap the benefits of physical exercise and we suggest that a reasonable program to begin with is to follow the minimum recommended international guidelines on exercise for healthy adults, i.e., at least 150 min of moderate intensity exercise a week (30). As MG by its nature can involve fluctuations in symptoms dependent or independent of physical exercise, patients should always contact their physician if experiencing sustained worsening of symptoms, to receive supportive advice on further management.

Thus, based on the current knowledge described in this review we propose that stable MG patients are encouraged to perform physical exercise. However, it remains to be determined to what extent physical exercise should form part of routine treatment regimens in MG and if there are any specific training protocols of particular benefit to individuals with the disease. In order to establish tailored training recommendations for MG patients, further studies are warranted. To improve the impact of such studies the recommended MG outcome measures for clinical MG trials should be used (34). However, this set of recommendations does not specifically address exercise studies and, as illustrated by the studies in this review, there remains a lack of consensus on what outcome measures of fatigue and physical performance status should be used. It would be desirable to have a fixed battery of validated outcome measures customized for MG trials, that cover disease activity and QoL as well as physical performance status and measures of physical and mental fatigue.

A unified taxonomy of fatigue has been proposed for neurological diseases, which may be useful in future research studies to differentiate between performance fatigability and fatigue perception (18).

We await with interest the results of ongoing trials on the topic of exercise in MG (61) (NCT01047761), and hope that future research in this area will inspire even clearer guidelines on exercise for MG patients and their caregivers.

Exercise is a self-modifiable lifestyle factor which plays a vital role in preventing the development of a range of chronic diseases from potentially fatal illnesses such as cardiovascular disease to debilitating conditions such lumbago and fatigue. Based on the evidence documented in this review, we conclude that MG patients and their caregivers can be encouraged to commence tailored exercise programs in stable well-controlled MG, while bearing in mind that simply being more active and reducing overall sedentary time is just as important.

## Author Contributions

All authors contributed to manuscript drafting and revision, read and approved the submitted version.

## Conflict of Interest

The authors declare that the research was conducted in the absence of any commercial or financial relationships that could be construed as a potential conflict of interest.
